# Randomized, open-label phase II study of brigatinib and carboplatin plus pemetrexed and brigatinib alone for chemotherapy-naive patients with ALK-rearranged non-squamous non-small cell lung cancer: treatment rationale and protocol design of the B-DASH study (WJOG 14720 L)

**DOI:** 10.1186/s12885-023-11417-w

**Published:** 2023-09-25

**Authors:** Kazushige Wakuda, Hirotsugu Kenmotsu, Yuki Sato, Atsushi Nakamura, Hiroaki Akamatsu, Motoko Tachihara, Satoru Miura, Toshihide Yokoyama, Keita Mori, Kazuhiko Nakagawa, Nobuyuki Yamamoto

**Affiliations:** 1 Division of Thoracic Oncology, Shizuoka Cancer Center Hospital, 1007 Shimonagakubo Nagaizumi-Cho Suntou-Gun, Shizuoka, 411-8777 Japan; 2 Department of Respiratory Medicine, Kobe City Medical Center General Hospital, 2-1-1 Minatojimaminami-Machi Chuo-Ku Kobe, Hyogo, 650-0047 Japan; 3 Department of Pulmonary Medicine, Sendai Kousei Hospital, 4-15 Hirose-Cho Aoba-Ku Sendai, Miyagi, 980-0873 Japan; 4 Internal Medicine III, Wakayama Medical University, 811-1, Kimiidera, Wakayama, 641-8509 Japan; 5 Division of Respiratory Medicine, Department of Internal Medicine, Kobe University Graduate School of Medicine, 7-5-1 Kusunoki-Cho, Chuo-Ku, Kobe, Hyogo 650-0017 Japan; 6 Department of Internal Medicine, Niigata Cancer Center Hospital, 2-15-3 Kawagishi-Cho, Chuo-Ku, Niigata, 951-8566 Japan; 7 Department of Respiratory Medicine, Kurashiki Central Hospital, 1-1-1 Miwa, Kurashiki, Okayama 710-8602 Japan; 8 Dividion of Cliniccal Research Center, Shizuoka Cancer Center Hospital, 1007 Shimonagakubo Nagaizumi-Cho Suntou-Gun, Shizuoka, 411-8777 Japan; 9 Department of Medical Oncology, Faculty of Medicine, Kindai University, 377-2 Ohno-Higashi, Osaka-Sayama, Osaka, 589-8511 Japan

**Keywords:** Non-small cell lung cancer, ALK, Brigatinib, Pemetrexed, Chemotherapy

## Abstract

**Background:**

The ALTA-1L study compared brigatinib with crizotinib in untreated *ALK*-rearranged non-small cell lung cancer (NSCLC) patients, demonstrating the efficacy of brigatinib. Although the median progression-free survival (PFS) of brigatinib group was 24.0 months, the one-year PFS rate was 70%. In the NEJ009 study, patients with *EGFR* mutations showed improved outcomes with gefitinib plus chemotherapy compared with gefitinib monotherapy. To evaluate the efficacy of the combination of brigatinib with chemotherapy for patients with *ALK*-rearranged NSCLC, we designed B-DASH study (WJOG 14720L).

**Methods:**

B-DASH study is a multicenter, two-arm, phase II study. Eligible patients have untreated stage IIIB, stage IIIC, stage IV, or postoperative relapse *ALK*-rearranged nonsquamous NSCLC. Patients will be randomized in a 1:1 ratio to receive brigatinib (180 mg once daily with a 7-day lead-in period at 90 mg) monotherapy or carboplatin (area under the curve = 5 on day 1) plus pemetrexed (500 mg/m^2^ on day 1) and brigatinib in a 3-week cycle for up to four cycles, followed by pemetrexed and brigatinib as maintenance therapy. The target hazard ratio of 0.62 is set based on the NEJ009 study. With one-sided alpha = 0.20 and power = 0.8, the sample size for the B-DASH study was calculated to be 110, considering the possibility of patients dropping out. The primary endpoint is PFS. The key secondary endpoints are the overall response rate and overall survival. We will evaluate tumor-derived DNA from plasma specimens before treatment, 42 days after administering the study drug, and on the day of progressive disease. Recruitment began in November 2021 and is ongoing.

**Discussion:**

The efficacy of combination therapy with tyrosine kinase inhibitors and cytotoxic chemotherapy was demonstrated in patients with *EGFR* mutations but remains unclear in patients with ALK-rearranged NSCLC. The B-DASH study is the only trial of brigatinib combined with chemotherapy in patients with untreated *ALK*-rearranged NSCLC.

**Trial registration:**

jRCT identifier: jRCTs041210103.

## Background

Lung cancer is a leading cause of death worldwide. Non-small cell lung cancer (NSCLC) is a major pathological subtype of lung cancer. Many guidelines recommend that patients with stage IV NSCLC should be treated based on the presence of genetic alterations and expression of programmed death ligand 1 (PD-L1)[1–4]. Patients with anaplastic lymphoma kinase (*ALK*) fusion gene rearrangement account for 2–4% of lung adenocarcinoma [5, 6]. ALK tyrosine kinase inhibitors (TKI) are effective in patients with *ALK*-positive lung cancer. Alectinib, brigatinib, and lorlatinib are commonly used as first-line chemotherapy for patients with *ALK*-positive NSCLC based on the results of a phase III trial comparing crizotinib with new-generation ALK-TKI [7–10].

Brigatinib is a second-generation ALK-TKI. The results of the ALTA-1L trial, which compared crizotinib with brigatinib, showed that brigatinib significantly improved progression-free survival (PFS) (12-month PFS was 67% in the brigatinib group and 43% in the crizotinib group; hazard ratio (HR) = 0.49; 95% confidence interval (CI) = 0.33–0.74); *p* < 0.001) [9]. The final results of ALTA-1L were reported in 2021. Median PFS was 24.0 and 11.1 months in brigatinib and crizotinib groups, respectively [11]. Although brigatinib is effective as first-line chemotherapy for patients with *ALK*-positive NSCLC, approximately 30% of patients relapse at 12 months. Therefore, we need a more effective strategy for patients with *ALK* fusion gene rearrangements.

A study comparing epidermal growth factor receptor (EGFR)-TKI monotherapy with EGFR-TKI combined with cytotoxic chemotherapy has been reported in patients with *EGFR*mutations. The NEJ 009 trial, which compared gefitinib monotherapy (monotherapy group) with gefitinib combined with carboplatin plus pemetrexed (combination group), was reported in 2019 [12]. The study hierarchically analyzed PFS, progression-free survival 2 (PFS2), and overall survival (OS) as co-primary endpoints. Although there were no significant differences in PFS2 and OS between the two groups, PFS was significantly prolonged in the combination group (median PFS was 20.9 and 11.2 months in the combination and monotherapy groups, respectively; HR = 0.49, 95% CI = 0.39–0.62; *p* < 0.001). And overall response rate (ORR) was improved in the combination group compared with the monotherapy group. In 2022, the updated results of NEJ009 were reported. The median survival time was 38.5 months and 49.0 months in the monotherapy group and the combination group, respectively (HR = 0.82. 95% CI = 0.62–1.06; *p* = 0.127) [13]. Although the rate of grade ≥ 3 adverse events was higher in the combination group, there was no significant difference in the incidence of protocol treatment discontinuation due to adverse events between the two groups. A phase III trial with the same design was carried out in India and reported in 2020, showing that the addition of chemotherapy to gefitinib significantly prolonged PFS and OS. The ORR was also improved in combination therapy compared with gefitinib monotherapy [14]. The efficacy of the EGFR-TKI combined with the cytotoxic chemotherapy treatment strategy was reproducible. This treatment strategy will be tested in patients with *EGFR* mutations, and the FLAURA 2 study comparing osimertinib monotherapy to osimertinib combined with cytotoxic chemotherapy is ongoing. However, few trials assessing the efficacy of ALK-TKI plus chemotherapy have been performed for patients with ALK-positive NSCLC. Although the S1300 study (NCT02134912) assessed the efficacy of crizotinib plus pemetrexed, patients with NSCLC that had progressed after crizotinib were eligible, not previously untreated patients. It was reported that greater depth of response was associated with longer PFS and OS for patients with NSCLC treated with ALK-TKI [15]. Because gefitinib plus chemotherapy improved ORR compared with gefitinib monotherapy in patients with *EGFR* mutations, we hypothesize that ALK-TKI plus chemotherapy improved ORR leading to prolong PFS as patients with *EGFR* mutations.

Subgroup analysis of the PROFILE 1007 study, which compared crizotinib with chemotherapy for previously treated patients with ALK-positive NSCLC, showed that median PFS was 4.2 and 2.6 months for pemetrexed and docetaxel, respectively [16]. And, a retrospective study also showed that *ALK*-positive NSCLC patients on pemetrexed had a significantly longer PFS than *EGFR* wild type or *KRAS*wild type patients [17]. These reports suggested that pemetrexed was more effective than other cytotoxic agents in patients with ALK-positive NSCLC. Since pemetrexed is a key drug for patients with ALK rearrangement, we must check its efficacy with other drugs.

Therefore, we designed the B-DASH study, which compares the efficacy of brigatinib monotherapy and brigatinib combined with carboplatin and pemetrexed in patients with *ALK*-positive NSCLC.

## Methods / design

### Study design

**Fig. 1:**
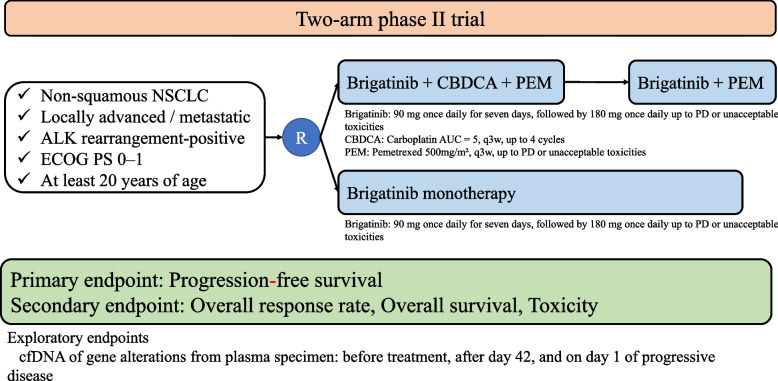
Study schema

Figure 1 shows an overview of the B-DASH study—a multicenter, two-arm, phase II study. Eligible patients will be randomized in a 1:1 ratio to receive brigatinib (monotherapy group) or brigatinib combined with carboplatin and pemetrexed (combined group). Randomization is stratified according to the disease stage (stage IIIB, IIIC, or IV vs. postoperative relapse) and brain metastasis (present vs. absent), because it is reported that these factors are prognostic factors. Patients in the monotherapy group will receive 180 mg of brigatinib orally once daily after 90 mg once daily for seven days as the lead-in period until disease progression and unacceptable toxicities. In the combined group, patients will receive four cycles of carboplatin (area under the curve, 5 on day 1) plus pemetrexed (500 mg/m^2^ on day 1) and brigatinib (same dose as in the monotherapy group), followed by pemetrexed and brigatinib as maintenance therapy.

Before registration in this trial, contrast-enhanced computed tomography (CT) of the chest and abdomen and contrast-enhanced magnetic resonance imaging (MRI) of the brain are required. CT and MRI will be performed every six weeks for 24 weeks after registration and every nine weeks after that.

The study is being conducted in compliance with the principles of the Declaration of Helsinki. Also. The study was approved by the central review board of the Shizuoka Cancer Center. This trial has been registered in the Japan Registry of Clinical Trials (jRCTs041210103).

### Eligibility criteria

The primary patient inclusion and exclusion criteria are presented in Table 1. Patients with untreated *ALK*-rearranged nonsquamous NSCLC are eligible for the study. *ALK*-rearrangement is diagnosed on the basis of locally approved *ALK* testing in Japan, such as immunohistochemistry, fluorescence in situ hybridization and next-generation sequencing. Other inclusion criteria are as follows: stage IIIB, stage IIIC, stage IV, or postoperative relapse for which definitive radiotherapy is impossible; Eastern Cooperative Oncology Group (ECOG) performance status (PS) 0–1; patients aged ≥ 20 years; and patients with at least one measurable lesion based on RECIST v1.1. Baseline brain metastasis is permitted if the patient is asymptomatic.

**Table 1:** Key inclusion and exclusion criteria

Key Inclusion Criteria	
√	Written informed consent obtained from the patient
√	Patients aged at least 20 years at the time of informed consent
√	Histologically or cytologically confirmed non-squamous non-small cell lung cancer
√	Confirmed ALK rearrangement using companion diagnostics
√	Stage IIIB, stage IIIC, stage IV, or postoperative relapse for which definitive radiotherapy is impossible
√	At least one measurable lesion based on RECIST v1.1
√	No symptomatic brain metastases
√	No symptomatic superior vena cava syndrome
√	No spinal cord compression
√	Patients with non-squamous non-small cell lung cancer previously untreated with chemotherapy
√	The specified time period has elapsed since the treatment defined in protocol
√	ECOG performance status 0–1
√	Adequate organ function
Key Exclusion Criteria	
√	Presence of active double cancers (synchronous cancers and metachronous cancers with disease-free interval within 3 years)
√	Presence of active systemic infection or topical infection requiring surgical treatment
√	Presence of active hepatitis B or C
√	Pregnant or Lactating women
√	Men who do not intend to use contraception
√	Presence of symptomatic cerebrovascular diseases or past history of these within one year
√	Nausea, vomiting, or absorption impediments causing difficulty with administration of the trial drug
√	Presence of congestive heart failure and unstable angina or past history of myocardial infarction within one year
√	History of interstitial lung disease, drug-induced interstitial lung disease, and radiation pneumonitis requiring treatment by steroid
√	Patients with psychiatric disorder or mental symptoms
√	Calculated based on prednisone, requiring continuous full-body administration of high-dosage steroids over 10 mg/day or currently using other immunosuppressant drugs
√	History of hypersensitivity to brigatinib, carboplatin, pemetrexed, and any excipients of these drugs
√	Other patients determined unfit by an attending physician will also be excluded

Abbreviations: *ALK* Anaplastic lymphoma kinase, *RECIST* Response Evaluation Criteria in Solid Tumors, *ECOG*, Eastern Cooperative Oncology Group

### Study endpoints

The B-DASH study will evaluate the efficacy and safety of brigatinib combined with carboplatin and pemetrexed in patients with untreated *ALK*-rearranged NSCLC. The primary endpoint is progression-free survival assessed using the RECIST criteria. The secondary endpoints include the ORR, OS, and the rate of adverse events. To assess the resistance mechanism, we will analyze tumor-derived DNA from plasma specimens before treatment, 42 days after the administration of the study drug, and on the date of progressive disease. The tumor-derived DNA analysis will be performed with next generation sequencing using the AVENIO ctDNA Surveillance Kit (Roche Diagnostics, Basel, Switzerland).

### Statistical considerations

The primary endpoint of the B-DASH study is progression-free survival assessed using the RECIST criteria assessed by investigator. Progression-free survival is the time from randomization to confirmation of progressive disease or death, whichever occurs first. The sample size of B-DASH study is calculated based on the median PFS of brigatinib monotherapy and hazard ration. We set the median PFS of brigatinib monotherapy in this study of 24.0 months based on the results of ALTA-1L study. The target hazard ratio of 0.62 is set based on the NEJ009 study. With one-sided alpha = 0.20 and power = 0.8, the sample size for the B-DASH study was calculated to be 110, considering drop out rate as 5%.

## Discussion

The efficacy of combination therapy with tyrosine kinase inhibitors and cytotoxic chemotherapy was demonstrated in patients with *EGFR* mutations but remains unclear in patients with *ALK*-rearranged NSCLC. The B-DASH study is the only trial of brigatinib combined with chemotherapy in patients with untreated *ALK*-rearranged NSCLC.

## Data Availability

Not applicable.

## Abbreviations

NSCLC: Non-small cell lung cancer
PD-L1: Programmed death ligand 1
ALK: Anaplastic lymphoma kinase
TKI: Tyrosine kinase inhibitors
PFS: Progression-free survival
HR: Hazard ratio
CI: Confidence interval
EGFR: Epidermal growth factor receptor
OS: Overall survival
CT: Computed tomography
MRI: Magnetic resonance imaging
ECOG: Eastern Cooperative Oncology Group
PS: Performance status
DNA: Deoxyribonucleic acid
